# Increased peak detection accuracy in over-dispersed ChIP-seq data with supervised segmentation models

**DOI:** 10.1186/s12859-021-04221-5

**Published:** 2021-06-14

**Authors:** Arnaud Liehrmann, Guillem Rigaill, Toby Dylan Hocking

**Affiliations:** 1 Institut des Sciences des Plantes de Paris-Saclay (IPS2), Université Paris-Saclay, Université Evry, CNRS, INRAE, 91405 Orsay, France; 2 Laboratoire de Mathématiques et Modélisation d’Evry (LAMME), Université Paris-Saclay, Université Evry, CNRS, 91037 Evry, France; 3 School of Informatics, Computing, and Cyber Systems (SICCS), Northern Arizona University, 86011 Flagstaff, AZ USA

**Keywords:** ChIP-seq, Histone modifications, Over-dispersion, Peak calling, Multiple changepoint detection, Likelihood inference, Supervised learning

## Abstract

**Background:**

Histone modification constitutes a basic mechanism for the genetic regulation of gene expression. In early 2000s, a powerful technique has emerged that couples chromatin immunoprecipitation with high-throughput sequencing (ChIP-seq). This technique provides a direct survey of the DNA regions associated to these modifications. In order to realize the full potential of this technique, increasingly sophisticated statistical algorithms have been developed or adapted to analyze the massive amount of data it generates. Many of these algorithms were built around natural assumptions such as the Poisson distribution to model the noise in the count data. In this work we start from these natural assumptions and show that it is possible to improve upon them.

**Results:**

Our comparisons on seven reference datasets of histone modifications (H3K36me3 & H3K4me3) suggest that natural assumptions are not always realistic under application conditions. We show that the unconstrained multiple changepoint detection model with alternative noise assumptions and supervised learning of the penalty parameter reduces the over-dispersion exhibited by count data. These models, implemented in the R package *CROCS* (https://github.com/aLiehrmann/CROCS), detect the peaks more accurately than algorithms which rely on natural assumptions.

**Conclusion:**

The segmentation models we propose can benefit researchers in the field of epigenetics by providing new high-quality peak prediction tracks for H3K36me3 and H3K4me3 histone modifications.

**Supplementary Information:**

The online version contains supplementary material available at 10.1186/s12859-021-04221-5.

## Background

Chromatin immunoprecipitation followed by high-throughput sequencing (ChIP-seq) is amongst the most widely used methods in molecular biology [15]. This method aims to identify transcription factor binding sites [20, 22] or post-translational histone modifications [24, 25], referred to as histone marks, underlying regulatory elements. Consequently, this method is essential to deepen our understanding of transcriptional regulation. The ChIP-seq assay yields a set of DNA sequence reads which are aligned to a reference genome and then counted at each genomic position. This results in a series $Y = (y_1,\dots ,y_n)$ of *n* non-negative integer count data $(y_i \in {\mathbb {Z}}_+)$, hereafter called coverage profile, ordered along a chromosome. The binding sites or histone marks of interest appear as regions with high read density referred to as peaks in the coverage profile.

Since there is a biological interest in detecting these peaks, several methods, hereafter called peak callers (*c*), have been developed / adapted and used to filter out background noise and accurately identify the peak locations in the coverage profile. They take a coverage profile of length *n* and classify each base from it as a part of the background noise (0) or peak (1), i.e. $c:Y\rightarrow \{0,1\}^n$. Among these peak callers we can mention MACS [26] and HMCan [2], two heuristics which are computationally fast but typically accurate only for a specific pattern, i.e. respectively sharp and broad peaks [7]. More recently, it has been proposed to solve the peak detection problem using either optimal constrained or unconstrained multiple changepoint detection methods [8, 12]. The constraints ensure that the segmentation model can be interpreted in terms of peaks and background noise which is a practitioner’s request. The unconstrained one doesn’t have an output segmentation with a straightforward interpretation in terms of peaks and needs to be followed by an ad-hoc post-processing rule to infer the start and end of peaks (see Fig. 2). For each of these methods, there are one or more tuning parameters that need to be set before solving the peak detection problem and that may affect the results accuracy.

In a supervised learning approach, Hocking et al. [7] introduced seven labeled histone mark datasets that are composed of samples from two different ChIP-seq experiments directed at histone modifications H3K36me3 and H3K36me3. In a recent study, after training different peak callers using these datasets, Hocking et al. [12] compared them and showed that the constrained segmentation model with count data following a Poisson distribution outperforms standard bioinformatics heuristics and the unconstrained segmentation model on these datasets.

### Modeling question

From a modeling perspective the constrained segmentation model and the Poisson noise are certainly the most natural assumptions to detect peaks in coverage profiles. However, it is not clear that they are realistic:

- By looking at the shapes of the peaks in coverage profiles (see for instance in Fig. 1), we can see that the background noise and the top of the peaks are sometimes separated by one or more subtle changes. In contrast to the constrained segmentation model, the unconstrained one should be able to capture these subtle changes. One major issue is that the output segmentation of the unconstrained model does not have a straightforward interpretation in terms of peaks.
- Parametric models such as the negative binomial [14, 17] or the Gaussian, following a proper transformation of the count data for the latter [1, 13], are preferred over the Poisson one for the analysis of many high-througput sequencing datasets. Indeed, count data often exhibit more variability than the Poisson model expects which changes the interpretation of the model and makes it difficult to estimate its parameters. These alternative parametric models are well known to reduce this phenomenon, also called over-dispersion.

In this work we try to start from these natural assumptions and show that it is possible to improve upon them.

### Contribution

1. We show that the distribution of counts from H3K36me3 and H3K4me3 datasets exhibits over-dispersion which invalidates the Poisson assumption. The two alternative noise models we propose (negative binomial with constant dispersion parameter & Gaussian after Anscombe transformation) effectively reduce the over-dispersion on these datasets (see Fig. 4).
2. We propose a new and rather natural post-processing rule to predict the start and end of peaks in an estimated unconstrained segmentation (see Fig. 2). Indeed, in the unconstrained segmentation we can observe several up (respectively down) changes and it is not obvious which one should be considered as the start or end of the peak. We show that this new post-processing rule improves the accuracy of the unconstrained segmentation model in both H3K36me3 and H3K4me3 datasets compared to the same model with previous rules described by Hocking et al. [12] (see Fig. 5).
3. Hocking et al. [11] described a procedure to extract all optimal constrained segmentations for a range of peaks. It is an essential internal step in the supervised approach for learning the penalty parameter of segmentation models. In this work we generalize this procedure so that it works with the unconstrained segmentation model and the post-processing rule mentioned in the previous point (see Algorithm 1).
4. We describe a method to learn jointly both the penalty and dispersion parameters of segmentation models with a negative binomial noise. We then compare the accuracy of unconstrained and contrained segmention models with different noise distributions on the labeled H3K36me3 and H3K4me3 datasets (see Fig. 6).

## Methods

### Segmentation models for ChIP-seq data

**Fig. 1:**
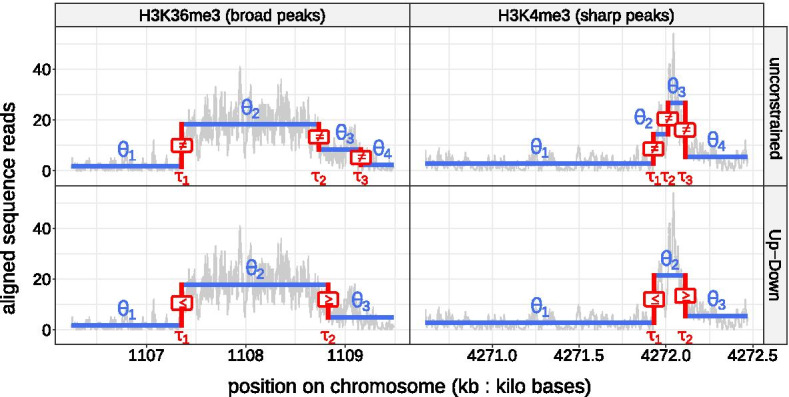
Examples of ChIP-seq coverage profiles from the histone mark H3K36me3 and H3K4me3 datasets. (**Top**) In blue  , a piecewise constant function affected by three unconstrained abrupt changes shown in red  . (**Bottom**) In blue  , a piecewise constant function affected by two constrained abrupt changes shown in red

#### Unconstrained segmentation model

The observed data $(y_1,\dots ,y_n)$ are supposed to be a realization of an independent random process $(Y_1,\dots ,Y_n)$. This process is drawn from a probability distribution ${\mathcal {F}}$ which depends on two parameters: $\theta$ is assumed to be affected by $K-1$ abrupt changes called changepoints and $\phi$ is constant. We denote $\tau _k$ the location of the *k*th changepoint with $k=\{1,\dots ,K-1\}$. By convention we introduce the fixed indices $\tau _0=0$ and $\tau _K=n$. The *k*th segment is formed by the observations $(y_{\tau _{k-1}+1} ,\dots , y_{\tau _k})$. $\theta _k$ stands for the parameter of the *k*th segment (see Fig. 1). Formally the unconstrained segmentation model [5], can be written as follows:

1 $$\begin{aligned} \forall i\;|\quad \tau _{k-1}+1 \le i \le \tau _{k},\quad Y_{i} \sim {\mathcal {F}}(\theta _k, \phi ). \end{aligned}$$

#### Constrained segmentation model

In order to have a segmentation model with a straightforward interpretation in terms of peaks, we add inequality constraints to the successive segment specific parameters $(\theta _{1},\dots ,\theta _{K})$ so that non-decreasing changes in these parameters are always followed by non-increasing changes. Therefore, we formally assume the following constrained segmentation model [8], hereafter called Up–Down:

2 $$\begin{aligned} \forall i\; |\quad \tau _{k-1}+1 \le i \le \tau _{k},\quad Y_{i} \sim {\mathcal {F}}(\theta _k, \phi )\quad \nonumber \\ {\text {subject to}} {\left\{ \begin{array}{ll} \theta _{k-1} \le \theta _{k} \quad \forall k \in \{2,4,\dots \} \\ \theta _{k-1} \ge \theta _{k} \quad \forall k \in \{3,5,\dots \} \end{array}\right. }. \end{aligned}$$

#### Probability distributions

In the case of the Poisson distribution we have ${\mathcal {F}}(\theta _k, \phi ) = {\text {Pois}}(\Lambda _k, \phi =\emptyset )$ where $\Lambda _k$ stands for the mean and the variance of the *k*th segment. In the case of the Gaussian distribution we have ${\mathcal {F}}(\theta _k, \phi ) = {\mathcal {N}}(\mu _k,\sigma ^2)$ where $\mu _k$ is the mean of the *k*th segment and $\sigma ^2$ is the variance assumed constant across the segments. Also in this case, the non-negative integer count data have been transformed in real values $({\mathbb {Z}}_+ \rightarrow {\mathbb {R}}_+)$ through an Anscombe transformation $(\sqrt{Y+\frac{3}{8}})$ which is a useful variance-stabilizing transformation for count data following a Poisson distribution [1]. In the case of the negative binomial distribution we have ${\mathcal {F}}(\theta _k, \phi ) = {\text {NB}}(\mu _k,\phi )$ where $\mu _k$ is the the mean of the *k*th segment and $\phi$ is the dispersion parameter that needs to be learned on the data. In this parametrization $\sigma ^2_k$, the variance of the *k*th segment, is $\mu _k + \phi ^{-1}\mu _k^2$.

#### Optimization problems

In both unconstrained and constrained optimal multiple changepoint detection problems, the goal is to estimate the changepoint locations $(\tau _1,\dots ,\tau _{K-1})$ and the parameters $(\theta _1,\dots ,\theta _{K})$ both resulting from the segmentation. Runge et al. [19] introduced *gfpop* (Graph-Constrained Functional Pruning Optimal Partitioning), an algorithm that solves both problems using penalized maximum likelihood inference. It implements several loss functions including the Gaussian, Poisson and negative binomial that allowed us to compare different noise models for the count data. The number of changepoints in a coverage profile being unknown, *gfpop* takes a non-negative penalty $\lambda \in {\mathbb {R}}_+$ parameter that controls the complexity of the output segmentation. Larger penalty $\lambda$ values result in models with fewer changepoints. The extreme penalty values are $\lambda = 0$ which yields $n-1$ changepoints, and $\lambda = \infty$ which yields 0 changepoint. The time complexity of *gfpop* is empirically ${\mathcal {O}}(Vn\log (n))$. Intuitively, *V* stands for the number states you will need to encode your priors about the form of the output segmentation, e.g. with the Up–Down model at each time the signal can be a part of the background noise (Down) or a peak (Up). Consequently, the empirical time complexity of *gfpop* with the Up–Down model is ${\mathcal {O}}(2n\log (n))$ while with the unconstrained model it is ${\mathcal {O}}(n\log (n))$.

### Rules for inferring the start and end of peaks with the unconstrained segmentation model

**Fig. 2:**
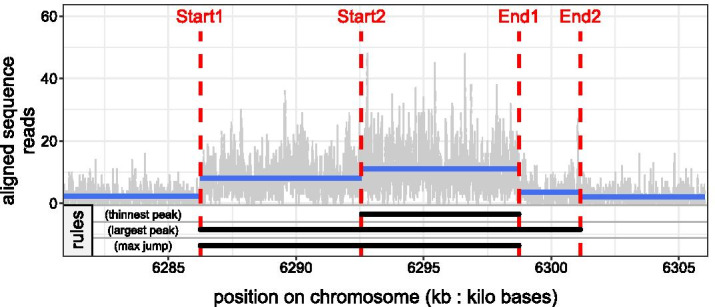
(**Top**) Segmentation of a coverage profile containing one peak using the unconstrained model. The location of the changepoints on the chromosome are shown by red dotted lines  . The mean of the segments are shown in blue  . According to this segmentation there are two alternative starts and two alternative ends of the peak, i.e. four alternative variants of the same peak formed by the regions: [Start1:End1], [Start1:End2], [Start2:End1] and [Start2:End2]. (**Bottom**) Three different rules are proposed to interpret the segmentation as peaks. *Thinnest peak*: the resulting peak is defined by the region [Start2:End1]. *Largest peak*: the resulting peak is defined by the region [Start1:End2]. *Max jump*: the resulting peak is defined by the region [Start1:End1]

As mentioned before, one of the main motivation of the Up–Down model is that it can be interpreted in terms of peaks which is a practitioner’s request. In the case of the unconstrained model, the output segmentation may results in successive non-decreasing changes (${\text {Up}}^*$), e.g. in Fig. 2: ${\text {Up}}^*=\{{\text {Start1}}, {\text {Start2}}\}$, and successive non-increasing changes (${\text {Dw}}^*$), e.g. in Fig. 2: ${\text {Dw}}^*=\{{\text {End1}}, {\text {End2}}\}$, in the signal. Thus, it is necessary to specify a post-processing rule to select the start and end of peaks among the returned changepoints in respectively each ${\text {Up}}^*$ and ${\text {Dw}}^*$. This results in $|{\text {Up}}^*| \times |{\text {Dw}}^*|$ alternatives of the same peak. *Rules.* We propose three different rules to select the start and end of peaks (see Fig. 2):

- *thinnest peak*: we select the last up change in ${\text {Up}}^*$ and the first down change in ${\text {Dw}}^*$ ;
- *largest peak rule*: we select the first up change in ${\text {Up}}^*$ and the last down change in ${\text {Dw}}^*$ ;
- *max jump*: we select the up and down change with the largest mean-difference in ${\text {Up}}^*$ and ${\text {Dw}}^*$.

Hocking et al. [12] introduced similar rules to the *thinnest peak* and *largest peak*.

### Labeled data for supervised learning peak detection

#### Tuning parameters

For each peak callers there are one or more tuning parameters that need to be set before solving the peak detection problem and that may greatly affect the result accuracy. For segmentation methods this parameter is the penalty $\lambda$ which controls the number of peaks in the resulting segmentation, while for heuristics, such as MACS or HMCan, they use a threshold parameter whose value allows to only consider the top *p* peaks given their significance. Moreover, if we want to model the over-dipersion phenomenon observed in the count data using a negative binomial probability distribution, this is done at the cost of another parameter $(\phi )$ that we need to set as well. Its value may also affect the number of peaks in the resulting segmentation. In theory, if the correct noise model was known, it would be possible to use statistical arguments to choose the parameter to use. However, in practice the correct noise model is complex and unknown. There are many factors that influence the signal and noise patterns in real ChIP-seq data, e.g. experimental protocols, sequencing machines, alignment software. These factors results in poor accuracy for the detection of peaks [7]. Therefore, we will consider the supervised peak detection problem in which the value of tuning parameters can be learned using manually determined labels that indicate a presence or absence of peaks.

**Fig. 3:**
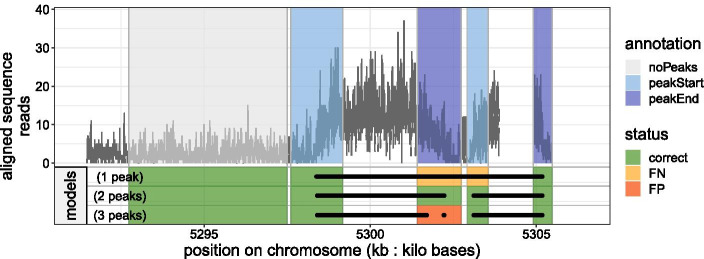
(**Top**) Example of a ChIP-seq coverage profile annotated by an expert biologist. The labels represented by colored rectangles indicate the absence  or presence of a peak, here characterized by its start  and its end  . (**Bottom**) The model with 1 peak in its output segmentation has an associated error of 2 ($2\times$ False Negative  ). The model with 3 peaks has an associated error of 1 ($1\times$ False Positive  ). The model with 2 peaks is a good model for which all the labels optimized on this coverage profile are correct

#### Benchmark datasets

Introduced by Hocking et al. [7], these seven labeled histone mark datasets are composed of samples from two different ChIP-seq experiments directed at modifications found on the histone 3 N-terminal tails. The first experiment is directed at histone H3 lysine 4 tri-methylation (H3K4me3), a modification localized in promoters. The second one is directed at histone H3 lysine 36 tri-methylation (H3K36me3), a modification localized in transcribed regions. Both these modifications are involved in the regulation of gene expression [21]. The histone modifications H3K4me3 and H3K36me3 are respectively characterized by sharp and broad peak patterns in coverage profiles. Expert biologists, with visual inspection, have annotated some regions by indicating the presence or absence of peaks. Then, they grouped the labels to form 2752 distinct labeled coverage profiles. Standard used for labeling by the expert biologists is described in Supplementary Text 1 of Hocking et al. [10].

#### Definition of labeled coverage profiles and errors

In the context of supervised peak detection each labeled coverage profile of size *n*, denoted $w\in {\mathbb {Z}}_+^n$, is a problem. Formally we have a set of *M* problems $(w_1,\dots ,w_M)$ where $M=2752$. Each problem $w_m$ is associated with a set of *N* labels $H_m = \{(s_1,e_1, h_1)\, \dots , (s_{N},e_{N}, h_{N})\}$ where *s* is the start genomic location of the label, *e* is the end genomic location of the label and *h* is the type of the label. There are four types of labels that allow some flexibility in the annotation (see Fig. 3):

- *noPeaks* label stands for a region that contains only background noise with no peak. If any peak is predicted in this region, the label counts as a false positive ;
- *peaks* label means there is at least one overlapping peak in that region. Hence, one or more peaks in that region is acceptable. If there is not at least one overlapping peak predicted in this region, it counts as a false negative ;
- *peakStart* and *peakEnd* labels stand for regions which should contain exactly one peak start or end. If more than one peak start / end is predicted in this region, the label counts as a false positive. Conversely, if less than one peak start / end is predicted in this region, the label counts as a false negative.

The set of labels $H_m$ is used to quantify the error $E_m$, i.e. the total number of incorrectly predicted labels (false positive $+$ false negative) in the coverage profile $w_m$ given the set of peaks returned by a peak caller.

### Supervised algorithms for learning tuning parameters of negative binomial segmentation models

#### Objective function

The error function for a given problem $w_m$, denoted $E_m:{\mathbb {R}}_+^2\rightarrow {\mathbb {Z_+}}$, is a mapping from the tuning parameters $(\phi , \lambda$) of negative binomial segmentation models to the number of incorrectly predicted labels in the resulting optimal segmentation. With the supervised peak detection approach the goal is to provide predictions of $\phi$ and $\lambda$ that minimize $E_m(\phi ,\lambda )$. The exact computation of the 2-dimensional defined $E_m(\phi ,\lambda )$ is intractable with respect to $\phi$. Thus, we computed it over 16 $\phi$ values evenly placed on the log scale between 1 and 10,000, $\Phi = (\phi _1=1,\dots ,\phi _{16}=10{,}000)$. Our results suggest that this grid of values is a good set of candidates to test in order to calibrate the dispersion parameter $\phi$ (see Additional file 1: Fig. 2). The exact computation of the error rate as a function of $\lambda$ ($\phi$ remains constant), a piecewise constant function, requires to retrieve all optimal segmentations up to 9 peaks. This way, on the advice of the biologists who annotated the benchmark datasets, we ensure that for each problem there is a segmentation with at least one false positive label and another with one false negative label. A procedure that retrieves one optimal segmentation for each targeted number of peaks $P^*$ has already been described by Hocking et al. [11]. It can be used with the Up–Down model for which there is at most one optimal segmentation that results in $P^*$ peaks but not with the unconstrained model for which there can be several ones. Indeed, the constraints in the Up–Down model require it to add, if the associated cost is optimal, 2 changepoints that lead to the formation of a new peak. With the unconstrained model adding a changepoint can either refine an already existing peak or, in combination with another changepoint, form a new peak. More generally there is a need of an algorithm that takes as input any penalized changepoint detection solver ${\mathcal {S}}$ with a penalty $\lambda$ constant along the changepoints, optionally the dispersion parameter $\phi$, and outputs all optimal segmentations between two peak bounds denoted ${\underline{P}}$ and ${\overline{P}}$. We present *CROCS* (Changepoints for a Range of ComplexitieS), an algorithm that meets this need.

#### Discussion of pseudocode

*CROCS* (*Algorithm* 1).**(i)** The algorithm begins by calling *SequentialSearch* [described underneath] to search two penalty bounds $\overline{\lambda }$ (line 6) and ${\underline{\lambda }}$ (line 5) that result in a segmentation with respectively ${\underline{P}}-1$ (line 3) and ${\overline{P}}+1$ (line 4) peaks. Indeed, using *gfpop* with the Up–Down model as solver ${\mathcal {S}}$, the number peaks in the resulting optimal segmentations is a non-increasing function of $\lambda$. This propriety guarantees that with the previous penalty bounds we can reach every optimal model from ${\underline{P}}$ to ${\overline{P}}$ peaks. For unconstrained segmentation models, we suspect it also should be true in the vast majority of cases. **(ii)** Then, the algorithm calls *CROPS* [described underneath] (line 7) to retrieve all the optimal segmentations between these two penalty bounds. **(iii)** Finally, a simple post-processing step (not shown in the algorithm) allows to remove segmentations with $P-1$ and $P+1$ peaks. The time complexity of the *CROCS* algorithm is bounded by the time complexity of the *CROPS* procedure, i.e. ${\mathcal {O}}({\mathcal {O}}({\mathcal {S}})(K_{{\underline{\lambda }}} - K_{\overline{\lambda }}))$, where $K_{\overline{\lambda }}$ and $K_{{\underline{\lambda }}}$ are the number of segments in optimal segmentations associated to respectively $\overline{\lambda }$ and ${\underline{\lambda }}$. ${\mathcal {O}}({\mathcal {S}})$ is the time complexity of the solver ${\mathcal {S}}$, e.g. empirically ${\mathcal {O}}(2n\log (n))$ for *gfpop* with the Up–Down model.

- *SequentialSearch* is a procedure described by Hocking et al. [11] that takes as input a problem $w_m$, a target number of peaks $P^*$ and outputs an optimal segmentation with $P^*$ peaks in addition to the penalty $\lambda$ for reaching it.
- *CROPS* is a procedure described by Haynes et al. [6] that takes as input a problem $w_m$, as well as two penalty bounds ${\underline{\lambda }}$ & $\overline{\lambda }$ and outputs all the optimal segmentations between these two bounds.

We slightly modified the original implementation of both *SequentialSearch* and *CROPS* in such way that they can work with any penalized changepoint detection solver ${\mathcal {S}}$ provided by the user.
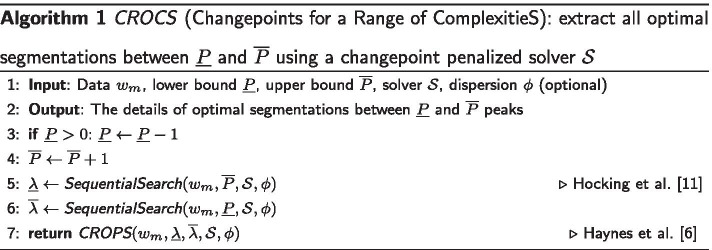


#### Learning jointly $\phi$ and $\lambda$

Once the error function $E_m(\phi \in \Phi ,\lambda )$ is computed for each problem of the training set, a natural way to learn the dispersion and penalty parameters is to select the pair of values $(\phi \in \Phi ,\lambda )$ that achieves the global minimum error. We denote these values $\phi ^*$ and $\lambda ^*$. Recall that $E_m(\phi \in \Phi ,\lambda )$ is piecewise constant on $\lambda$. The sum of $E_m(\phi \in \Phi ,\lambda )$ over all problems is still piecewise constant on $\lambda$. Therefore, $\phi ^*$ and $\lambda ^*$ can be easily retrieved using a sequential search. We refined the previous learning method, hereafter called *constant* $\lambda$, by taking advantage of the piecewise constant propriety of $E_m(\phi \in \Phi ,\lambda )$. Indeed, the minimum error is not reached for a unique penalty value $\lambda ^*$ but an interval denoted $I_{\lambda ,m}$. After fixing $\phi ^*$, we can use $I_{\lambda ,m}$ computed for each problem of the training set in order to learn a function that predicts problem-specific $\lambda$ values. This function is a solution of the interval regression problem described by Rigaill et al. [16]. We denote this learning method *linear* $\lambda$.

In the case of segmentation models with a Poisson or a Gaussian noise, the only tuning parameter that we need to learn is $\lambda$. Thus, the objective function becomes a 1-dimensional defined function denoted $E_m(\lambda )$. The methods we used to learn $\lambda$ are similar than those presented above (see Hocking et al. [12] for more details).

## Empirical results

### Cross-validation setup and evaluation metric

In the following section, for each model compared, a 10-fold or 4-fold^1^ cross-validation was performed on each of the seven datasets. Here, the results are shown by type of experiments (H3K36me3 & H3K4me3). The metric we used to evaluate the performance of our models is the test accuracy which can be formally written $1-\left( \sum _{m\in {\text { test set}}}E_m\ /\ \sum _{m\in {\text { test set}}} |H_m|\right)$. One may be concerned about the size of the datasets used for supervised learning of the tuning parameters. We have shown in Additional file 1: Fig. 1 that only a dozens of labels are enough to learn tuning parameters and associated segmentations close to the model-specific maximum accuracy. By increasing the number of labels in the learning set, the accuracy also becomes more consistent between test folds.

### Learning of tuning parameters

In previous section we have described two methods for learning the tuning parameters of segmentation models. Based on results shown in Additional file 1: Fig. 3, for the rest of this section, the parameters of the models compared on H3K36me3 datasets are learned through the *constant *$\lambda$ method. The parameters of the models compared on H3K4me3 datasets are them learned through the *linear* $\lambda$ method.

**Fig. 4:**
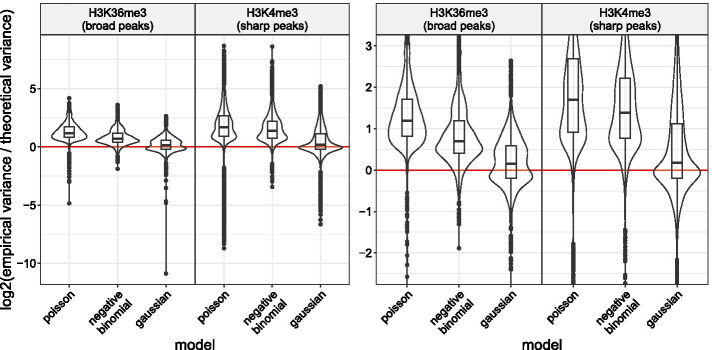
The over-dispersion exhibited by count data under a Poisson noise model can be effectively reduced using a negative binomial or a Gaussian transformed noise model. The red  indicator line stands for the equality of the theoretical and empirical variances. (**Left**) Observations above this line stand for over-dispersed count data. Observations under this line stand for under-dispersed count data. (**Right**) A zoom on the **Left** distributions

### The over-dispersion exhibited by count data under a Poisson noise model can be effectively reduced using a negative binomial or a Gaussian transformed noise model

Initially, we wanted to validate the presence of over-dispersion in count data following a Poisson distribution. In a second step, we wanted to confirm that alternative noise models such as the negative binomial or the Gaussian one, following an Anscombe transformation of the counts for the latter, could allow us to reduce this over-dispersion. A simple way to highlight the over-dispersion is to plot the $\log _2{\text {-ratio}}$ of the empirical and theoretical variances of count data. If the $\log _2{\text {-ratio}}$ is positive, the distribution of count data exhibits over-dispersion. If it is negative, the distribution of count data exhibits under-dispersion. If it is null, the dispersion of the count data does not show inconsistency with respect to the noise model. In Fig. 4, each observation corresponds to a segment from the segmentations selected during the cross-validation procedure for the 2752 coverage profiles. The segmentation were computed using *CROCS* with *gfpop* and the unconstrained model as solver. Then, We estimated the empirical and theoretical variances for each of the selected segments. In the case of the Poisson noise model, the estimated theoretical variance is formally written $\hat{\sigma _k}^2 = \hat{\mu }$, where $\hat{\mu }$ stands for the estimation of the mean of count data belonging to the same segment. For the negative binomial one it is formally written $\hat{\sigma }^2 = \hat{\mu }+\phi ^{-1}\hat{\mu }^2$, where $\phi$ stands for the dispersion parameter learned during the cross-validation procedure. For the Gaussian one, the theoretical variance is assumed constant across the segments. We estimated it using the mean squared error computed over all segments. In Fig. 4 we can see that in both H3K36me3 and H3K4me3 datasets the median of the $\log _2{\text {-ratio}}$ is above 1 with the Poisson noise model. Hence, For most observations the empirical variance is at least two times larger than the theoretical variance. Therefore, count data under the Poisson noise model shows a clear over-dispersion phenomenon. In both H3K36me3 and H3K4me3 datasets, the median of the $\log _2{\text {-ratio}}$ is slightly closest to 0 with the negative noise model than with Poisson noise one (from 1.19 to 0.70 in H3K36me3 and 1.69 to 1.39 in H3K4me3). Therefore, the negative noise model helps partially correct this over-dispersion. The reduction is even greater with the Gaussian transformed noise model (from 1.19 to 0.16 in H3K36me3 and 1.69 to 0.18 in H3K4me3).

### Max jump is the most accurate rule for inferring the peaks in segmentations obtained through the unconstrained model

**Fig. 5:**
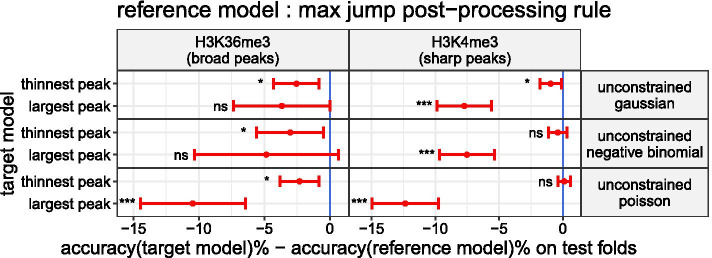
Max jump is the most accurate rule for inferring the peaks in segmentations obtained through a unconstrained model. The mean of differences in accuracy and its 95% CI computed on the test folds pooled by types of experiment (H3K36me3 & H3K4me3) are shown in red  . If the mean of differences in accuracy is negative (left side of the blue indicator line  ), the *max jump* rule is better in average than the target rule. The results of the paired t-test used to assess the difference of mean accuracy are summarized in the following way: non significant (ns) means adjusted p-value $>0.05$; * means adjusted p-value $\le 0.05$; *** means adjusted p-value $\le 0.001$

Solving the peak detection problem with the unconstrained model requires to introduce a rule for selecting the changepoints corresponding to the start and end of the peaks in the output segmentation. We wanted to compare the peak detection accuracy of the new rule we propose (*max jump*) against the others (*largest peak* & *thinnest peak*) which have an equivalence in Hocking et al. [12]. In the user guide of how to create labels in ChIP-seq coverage profiles [7], the authors strongly advise to label peaks which are obviously up with respect to the background noise. Hence, we expected that the *max jump* rule, which sets the start and end of the peaks on the change with the largest mean-difference, performs at least as well as the other two rules. In Fig. 5, we look at the mean of differences in accuracy between each model with either the *largest peak* or *thinnest peak* rule, denoted target models, against the same model with the *max jump* rule, denoted reference model. In agreement with our expectation, we observe that for the different models in both H3K36me3 & H3K4me3 datasets, the mean accuracy of the *max jump* rule is greater than the mean accuracy of the *largest peak* rule (3.66–12.36% more accurate on average). Except for the unconstrained model with a Poisson noise in H3K4me3 (0.11% less accurate on average), the mean accuracy of the *max jump* rule is also greater than the mean accuracy of the *thinnest peak* (0.38–3.03% more accurate on average). In order to test if the mean accuracy of the target and the reference models are significantly different, we performed a paired t-test. The accuracy of each fold were previously pooled by type of experiments as it is suggested in Fig. 5. After correcting the p-values of the paired t-test with the Benjamini & Hochberg method, eight differences were still significant (adjusted p-value $<0.05$). As a result of these observations, for the next comparisons we will infer the peaks in the output segmentations obtained with the unconstrained model using the new *max jump* rule we propose.

### The unconstrained model with a negative binomial or a Gaussian transformed noise is more accurate than previous state-of-the-art

**Fig. 6:**
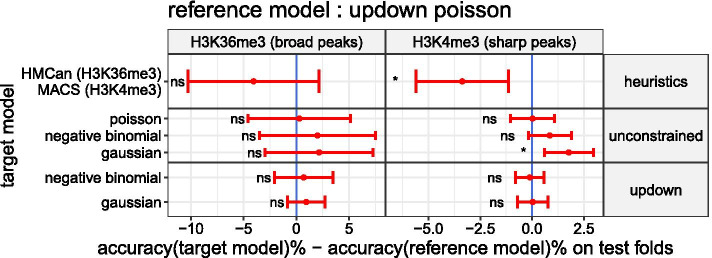
The unconstrained model with a negative binomial or a Gaussian transformed noise is more accurate than previous state-of-the-art. The mean of differences in accuracy and its 95% CI computed on the test folds pooled by types of experiment (H3K36me3 & H3K4me3) are shown in red  . If the mean of differences in accuracy is negative (left side of the blue indicator line  ), the Up–Down model with a Poisson noise is better in average than the target model. The results of the paired t-test used to assess the difference of mean accuracy are summarized in the following way: non significant (ns) means adjusted p-value $>0.05$; * means adjusted p-value $\le 0.05$

We wanted to compare the peak detection accuracy of the Up–Down model with a Poisson noise^2^ against other segmentation models such as the unconstrained or Up–Down model with either a negative binomial or a Gaussian transformed noise. HMCan, MACS and other heuristics have already been compared to the Up–Down model with a Poisson noise in Hocking et al. [12]. We included them again as a baseline from the bioinformatics literature. Both of them use a threshold that affects their peak detection accuracy and whose learning is also described in the previous cited study. Because we saw in previous results that a negative binomial or Gaussian transformed noise effectively reduces the over-dispersion exhibited by count data under a Poisson noise, we expected that the unconstrained or Up–Down model with these alternative noises will improve the peak detection accuracy on the test set. In Fig. 6 we look at the mean of differences in accuracy between the Up–Down model with a Poisson noise, denoted reference model, against other segmentation models and heuristics, denoted target models. In agreement with our expectation, we can see that the unconstrained model with a negative binomial noise has a mean accuracy greater than the reference model in both H3K36me3 and H3K4me3 datasets (respectively 2.0% and 0.86% more accurate on average). It has also a greater mean accuracy with a Gaussian transformed noise (respectively 2.15% and 1.77% more accurate on average). As described previously, in order to test if the mean accuracy of the target and the reference models are significantly different, we performed a paired t-test. After correcting the p-values, the unconstrained model with a Gaussian transformed noise was still significant (adjusted p-value $<0.05$). Note that the unconstrained model with a Poisson noise has a mean accuracy similar to reference model (the mean of differences in accuracy < 0.5% in both datasets). Thus, the improvement in accuracy cannot be attributed solely to the unconstrained model with the *max jump* rule but also to the distribution chosen for the noise. In disagreement with our expectation, with the Up–Down model the use of alternative noise distributions does not improve significantly the accuracy compared to the Poisson one (mean of differences in accuracy < 1% in H3K36me3 and < 0.1% in H3K4me3).

**Fig. 7:**
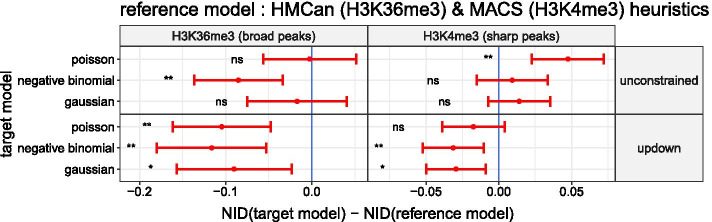
The Up–down segmentation models are more robust than the heuristics from the bioinformatics literature HMCan and MACS. The mean of differences of the normalized information distance (NID) and its 95% CI are shown in red  . If the mean of differences in NID is negative (left side of the blue indicator line  ), the target segmentation model is more robust in average than HMCan (H3K36me3) or MACS (H3K4me3). The results of the paired t-test used to assess the difference of mean of NID are summarized in the following way: non significant (ns) means adjusted p-value $>0.05$; * means adjusted p-value $\le 0.05$; ** means adjusted p-value $\le 0.01$

### The Up–down segmentation models are more robust than the heuristics from the bioinformatics literature HMCan and MACS

In addition to comparing the peak detection accuracy, we wanted to assess the robustness of segmentation models against the heuristics HMCan and MACS. To assess the robustness of the segmentation models and heuristics we used the coverage profiles from biological replicates available in each of the seven labeled histone mark datasets. The value of tuning parameters for the segmentation models and heuristics are the same as those learned during the cross-validation procedure. As explained in the introduction, the peak calling problem can be seen as a binary classification problem. In this framework each base from the coverage profiles are classified as a part of the background noise (0) or peak (1). Hence, the robustness can be assessed by computing the distance between partitions of the coverage profiles from the biological replicates. The more the distance between these partitions is close to zero the more the segmentation model or the heuristic is robust. As a metric we used the normalized information distance, or NID, which has a range between 0 and 1 [3, 23]. For each genomic chunk we computed the NID between all pairs of biological replicates. In Fig. 7 we look a the mean of differences of NID between segmentation models and the heuristics HMCan or MACS. We can see that the mean of the NID of Up–Down models, independently of the noise model, is lower than with the heuristics HMcan and MACS in both H3K36me3 and H3K4me3 datasets (respectively from 0.09 to 0.12 and 0.02 to 0.03 less distant on average). After correcting the p-values of the paired t-test with the Benjamini & Hochberg method, five differences were still significant (adjusted p-value $<0.05$). Regarding the unconstrained models, except for the negative binomial noise model in the H3K24me3 datasets (NID is lower by 0.09 in average & paired t-test with adjusted p-value $<0.01$), there is no clear improvement in robustness compared to the heuristics HMCan or MACS. With the Poisson model, which do no reduce the over-dispersion, we conclude even the opposite in the H3K4me3 datasets (NID is longer by 0.05 in average, paired t-test with adjusted p-value $<0.01$).

## Discussion

### Modeling of over-dispersed ChIP-seq count data

We have seen in Fig. 4 that count data under a Poisson noise model exhibit over-dispersion in H3K36me3 and H3K4me3 datasets. We have shown that this over-dispersion can be effectively reduced in these datasets using either a negative binomial or a Gaussian transformed noise model.

The use of a negative binomial noise model implies that we must be able to estimate a suitable value for the $\phi$ dispersion parameter. We have proposed to learn it jointly with the penalty of the segmentation model directly on the labeled coverage profiles. More precisely, a constant $\phi$ is selected because it minimizes the label errors of the training set. The negative binomial combined with the constant dispersion parameter allows the phenomenon of over-dispersion to be slightly reduced.

With the Gaussian noise model there are no additional parameters than the penalty of the segmentation model to set. This is an advantage compared to the negative binomial one. In this study, in order to satisfy the Gaussian proprieties, we transformed the count data with an Anscombe transformation which is highly appreciated for its variance stabilization properties. Gaussian transformed noise model allowed to reduce the over-dispersion even more efficiently than the negative binomial noise model on the H3K4me3 and H3K36me3 datasets, while being simpler to implement.

### Segmentation models for peak detection in ChIP-seq count data

The unconstrained model seems to capture more subtle changes in count data than the Up–Down one which have sometimes a poor fit to the signal (see Fig. 1). One major issue of the unconstrained model is its output segmentation which doesn’t have a straightforward interpretation in terms of peaks compared to the Up–Down one. The introduction of the *max jump* rule (see Fig. 2), which have shown to perform at least as well as rules proposed in Hocking et al. [12] (*thinnest peak* & *largest peak*), helps to correct this weakness (see Fig. 5).

In Fig. 6 we have seen that when combining the unconstrained model with a negative binomial or a Gaussian transformed noise it is possible to improve upon the natural and current state-of-the-art on the peak detection accuracy, the Up–Down model with a Poisson noise, in both H3K36me and H3K4me3 datasets. We argue that this improvement is likely explained by the ability of the negative binomial and the Gaussian transformation to reduce the over-dispersion as illustrated in Fig. 4. In summary, we believe that the better we model dispersion the better we improve the accuracy of the segmentation model. Figure 7 have shown that the unconstrained segmentation model with noise models reducing over-dispersion are also at least as robust as MACS or HMCan heuristics. It is an important criterion showing the applicability of our proposed models.

Still in Fig. 6, we have seen that the Up–Down model with a negative binomial or a Gaussian transformed noise, which reduce the over-dispersion phenomenon, doesn’t improve the accuracy upon the Up–Down model with a Poisson noise. One hypothesis to explain these results is that the constraints, which lead to the reduction of the space of optimal reachable segmentations with the Up–Down model, also reduce the probability of adding biologically uninformative changepoints induced by the over-dispersion. Consequently, the Up–Down model has the advantage to be a model with good internal over-dipsersion resistance properties but is bounded by its poor adaptability to the signal. We argue the constraints also explain that the Up–Down model is more robust than the unconstrained model and the MACS and HMcan heuristics (see Fig. 7).

We have added several supplementary figures (see Additional file 1: Figs. 4–10) which illustrate typical results from the test folds for the MACS and HMCan heuristics as well as our proposed segmention models.

### Segmentation models applied to other types of ChIP-seq experiments

In this paper, the broad (H3K36me3) and sharp (H3K4me3) histone signals have been discussed. Previous studies already demonstrated the applicability of optimal changepoint algorithms to other types of experiment. For example, Fig. 7 in Hocking and Bourque [9] showed that optimal changepoint algorithms on H3K9me3 and H3K27me3 data typically result in peaks with intermediate sizes (3.5–3.9 kb on average) compared with the relatively small H3K4me3 (1.0–1.7 kb) and relatively large H3K36me3 (35.8–48.0 kb). The peak calling of transcription factor binding sites such as MAX, SRF and NRSF was also previously tested (see Supplementary Fig. 3 in Hocking et al. [7]). By reducing the over-dispersion in count data with the Gaussian transformed or the negative binomial noise models, we would expect similar improvements in accuracy for these other experiment types. Furthermore, we did not test our proposed models on mixed signal like Pol II. We leave the two last points for future research.

## Conclusion

We developed the *CROCS* algorithm that computes all optimal models between two peak bounds, given any segmentation algorithm with constant penalty $\lambda$ for each changepoint. This set of optimal segmentations is essential to compute the error rate function, which is in turn used in the supervised approach for learning the tuning parameters of the segmentation models. We proposed to solve the peak detection problem by using the unconstrained segmentation model that takes advantage of the *max jump* rule we introduced as well as the negative binomial or Gaussian transformed noise model. We have shown that this model improves upon the accuracy of the model built on natural assumptions (constrained segmentation (Up–Down) with Poisson noise model) in both H3K36me3 and H3K4me3 datasets. The unconstrained model with the negative binomial or Gaussian transformed noise model can be used to provide new high-quality peak prediction tracks for H3K36me3 and H3K4me3 histone modifications. These peak prediction tracks will be a more accurate reference for researchers in the field of epigenetics who want to analyze these data.

### Future work

Our results suggest that with both negative binomial and Gaussian transformed noise models the over-dispersion could be further reduced. Regarding the negative binomial noise model, one could think about predicting a local dispersion parameter for each coverage profile. Furthermore, the literature about Gaussian transformations is wide and a comparative study integrating segmentation models with different transformations for count data, e.g. the Box–Cox transformation, arcsin square root transformation or log-transformation, would also be an interesting avenue for future work. As described in Anscombe [1] some of these well-known transformations have, in theory, better variance-stabilizing proprieties for over-dispersed count data than the one we used in this study $(\sqrt{Y+\frac{3}{8}})$. Still, they are highly dependent on the estimation of the dispersion parameter $\phi$ which in our case can be directly taken into account in the statistical model, i.e by using the negative binomial noise model implemented in *gfpop*.

In this paper we explored two different segmentation models, the unconstrained segmentation model and a constrained segmentation model where each non-decreasing change is followed by an non-increasing change in the mean (Up–Down). The *gfpop* method makes it possible to model changepoints even more precisely by constraining for example the minimum size of jumps or the minimum size of segments. It would be interesting in future work to test other constrained models or to model the auto-correlation [4, 18] in the context of the peak detection problem in ChIP-seq data.

## Supplementary Information

**Additional file 1**. Supplementary materials for “Increased peak detection accuracy in over-dispersed ChIP-seq data with supervised segmentation models”. 

## Data Availability

The labeled histone mark data are available here: https://rcdata.nau.edu/genomic-ml/chip-seq-chunk-db/. The scripts used to compute the models and analyse the results are available in the “aLiehrmann/chip_seq_segmentation_paper” *GitHub* repository: https://github.com/aLiehrmann/chip_seq_segmentation_paper. A reference implementation of the *CROCS* algorithm is available in the R package of the same name: https://github.com/aLiehrmann/CROCS. The package’s vignette describes the supervised learning procedure and the user can easily adapt the code to his own data.

## Abbreviations

ChIP-seq: Chromatin immunoprecipitation followed by high-throughput sequencing
Up–Down: Constrained segmentation model where each non-decreasing change is followed by an non-increasing change in the mean
gfpop: Graph-constrained functional pruning optimal partitioning
NID: Normalized information distance

## Footnotes

1 In order to satisfy the assumption about the independence between the training and test set in the cross-validation, we could not exceed 4-fold in two of the seven benchmark datasets (for more details see caption of Additional file 1: Table 1).

2 Model built on natural assumptions to detect peaks in coverage profiles and actual state-of-the-art on H3K36me3 and H3K4me3 datasets.

## References

[1] Anscombe FJ (1948). The transformation of poisson, binomial and negative-binomial data. Biometrika.

[2] Ashoor H, Herault A, Kamoun A, Radvanyi F, Bajic VB, Barillot E, Boeva V (2013). Hmcan: a method for detecting chromatin modifications in cancer samples using chip-seq data. Bioinformatics.

[3] Chiquet J, Rigaill G, Sundqvist M. Aricode: efficient computations of standard clustering comparison measures (2020). https://CRAN.R-project.org/package=aricode

[4] Cho H, Fryzlewicz P (2015). Multiple-change-point detection for high dimensional time series via sparsified binary segmentation. J R Stat Soc Ser B (Statistical Methodology).

[5] Cleynen A, Lebarbier E (2014). Segmentation of the poisson and negative binomial rate models: a penalized estimator. ESAIM Prob Stat.

[6] Haynes K, Eckley IA, Fearnhead P. Computationally efficient changepoint detection for a range of penalties (2017) 10.1007/s11222-016-9687-5 PMC6994226 32063685

[7] Hocking TD, Goerner-Potvin P, Morin A, Shao X, Pastinen T, Bourque G (2017). Optimizing chip-seq peak detectors using visual labels and supervised machine learning. Bioinformatics.

[8] Hocking T, Rigaill G, Bourque G (2015). Peakseg: constrained optimal segmentation and supervised penalty learning for peak detection in count data. Proc Mach Learn Res.

[9] Hocking TD, Bourque G (2020). Machine learning algorithms for simultaneous supervised detection of peaks inmultiple samples and cell types. Pac Symp Biocomput.

[10] Hocking TD, Rigaill G, Fearnhead P, Bourque G. A log-linear time algorithm for constrained changepoint detection. arXiv:1703.03352 (2017)

[11] Hocking TD, Rigaill G, Fearnhead P, Bourque G. Generalized functional pruning optimal partitioning (GFPOP) for constrained changepoint detection in genomic data. arXiv:1810.00117 (2018)

[12] Hocking TD, Rigaill G, Fearnhead P, Bourque G (2020). Constrained dynamic programming and supervised penalty learning algorithms for peak detection in genomic data. J Mach Learn Res.

[13] Law CW, Chen Y, Shi W, Smyth GK. voom: precision weights unlock linear model analysis tools for rna-seq read counts. Genome Biol. 2014;15. 10.1186/gb-2014-15-2-r29 PMC4053721 24485249

[14] Love M, Huber W, Anders S. Moderated estimation of fold change and dispersion for rna-seq data with deseq2. Genome Biol. 2014;15. 10.1186/s13059-014-0550-8 PMC4302049 25516281

[15] Marinov GK (2018). A decade of chip-seq. Brief Funct Genom.

[16] Rigaill G, Hocking T, Vert J-P, Bach F (2013). Learning sparse penalties for change-point detection using max margin interval regression. Proc Mach Learn Res.

[17] Robinson MD, McCarthy DJ, Smyth GK (2010). edger: a bioconductor package for differential expression analysis of digital gene expression data. Bioinformatics.

[18] Romano G, Rigaill G, Runge V, Fearnhead P. Detecting abrupt changes in the presence of local fluctuations and autocorrelated noise. arXiv:2005.01379 (2020)

[19] Runge V, Hocking TD, Romano G, Afghah F, Fearnhead P, Rigaill G. gfpop: an R package for univariate graph-constrained change-point detection. arXiv:2002.03646 (2020)

[20] Schmidt D, Wilson MD, Ballester B, Schwalie PC, Brown GD, Marshall A, Kutter C, Watt S, Martinez-Jimenez CP, Mackay S, Talianidis I, Flicek P, Odom DT. Five-vertebrate chip-seq reveals the evolutionary dynamics of transcription factor binding. Science. 2010;1036–1040. 10.1126/science.1186176 PMC3008766 20378774

[21] Sims RJ, Nishioka K, Reinberg D (2003). Histone lysine methylation: a signature for chromatin function. Trends Genet.

[22] Valouev A, Johnson DS, Sundquist A, Medina C, Anton E, Batzoglou S, Myers RM, Sidow A (2008). Genome-wide analysis of transcription factor binding sites based on chip-seq data. Nat Methods.

[23] Vinh NX, Epps J, Bailey J (2010). Information theoretic measures for clusterings comparison: variants, properties, normalization and correction for chance. J Mach Learn Res.

[24] Young MD, Willson TA, Wakefield MJ, Trounson E, Hilton DJ, Blewitt ME, Oshlack A, Majewski IJ. Chip-seq analysis reveals distinct h3k27me3 profiles that correlate with transcriptional activity. Nucl Acids Res. 2011;7415–7427. 10.1093/nar/gkr416 PMC3177187 21652639

[25] Zhang B, Zheng H, Huang B, Li W, Xiang Y, Peng X, Ming J, Wu X, Zhang Y, Xu Q, Liu W, Kou X, Zhao Y (2016). Allelic reprogramming of the histone modification h3k4me3 in early mammalian development. Nature.

[26] Zhang Y, Liu T, Meyer CA, Eeckhoute J, Johnson DS, Bernstein BE, Nusbaum C, Myers RM, Brown M, Li W, Liu XS. Model-based analysis of chip-seq (macs). Genome Biol. 2008;9. 10.1186/gb-2008-9-9-r137 PMC2592715 18798982

