# Supplementary material for: Increased peak detection accuracy in over-dispersed ChIP-seq data with supervised segmentation models
Source: BMC Bioinformatics. 2021 Jun 14;22:323. doi: 10.1186/s12859-021-04221-5 (PMC8201703; doi:10.1186/s12859-021-04221-5)
Supplement: Supplementary file 1 — Additional file 1. Supplementary materials for “Increased peak detection accuracy in over-dispersed ChIP-seq data with supervised segmentation models”. [file 12859_2021_4221_MOESM1_ESM.pdf]

Supplementary materials for "Increased peak detection accuracy in over-dispersed ChIP-seq data with supervised segmentation models"

datasets	type of ChIP-Seq experiment	number of folds
H3K36me3_AM_immune	H3K36me3 (broad peaks)	10
H3K36me3_TDH_immune	H3K36me3 (broad peaks)	4
H3K36me3_TDH_other	H3K36me3 (broad peaks)	4
H3K4me3_PGP_immune	H3K4me3 (sharp peaks)	10
H3K4me3_TDH_immune	H3K4me3 (sharp peaks)	10
H3K4me3_TDH_other	H3K4me3 (sharp peaks)	10
H3K4me3_XJ_immune	H3K4me3 (sharp peaks)	10

Table 1: Summary of the number of folds in the cross-validation procedure by dataset. Two of the seven labeled histone mark datasets, i.e. H3K36me3_TDH_immune & H3K36me3_TDH_other, can be considered small datasets as they include biological replicates from four independent genomic chunks. In order to satisfy the assumption of independence between the training and test set in the cross-validation, we could not exceed 4-fold for both of them.

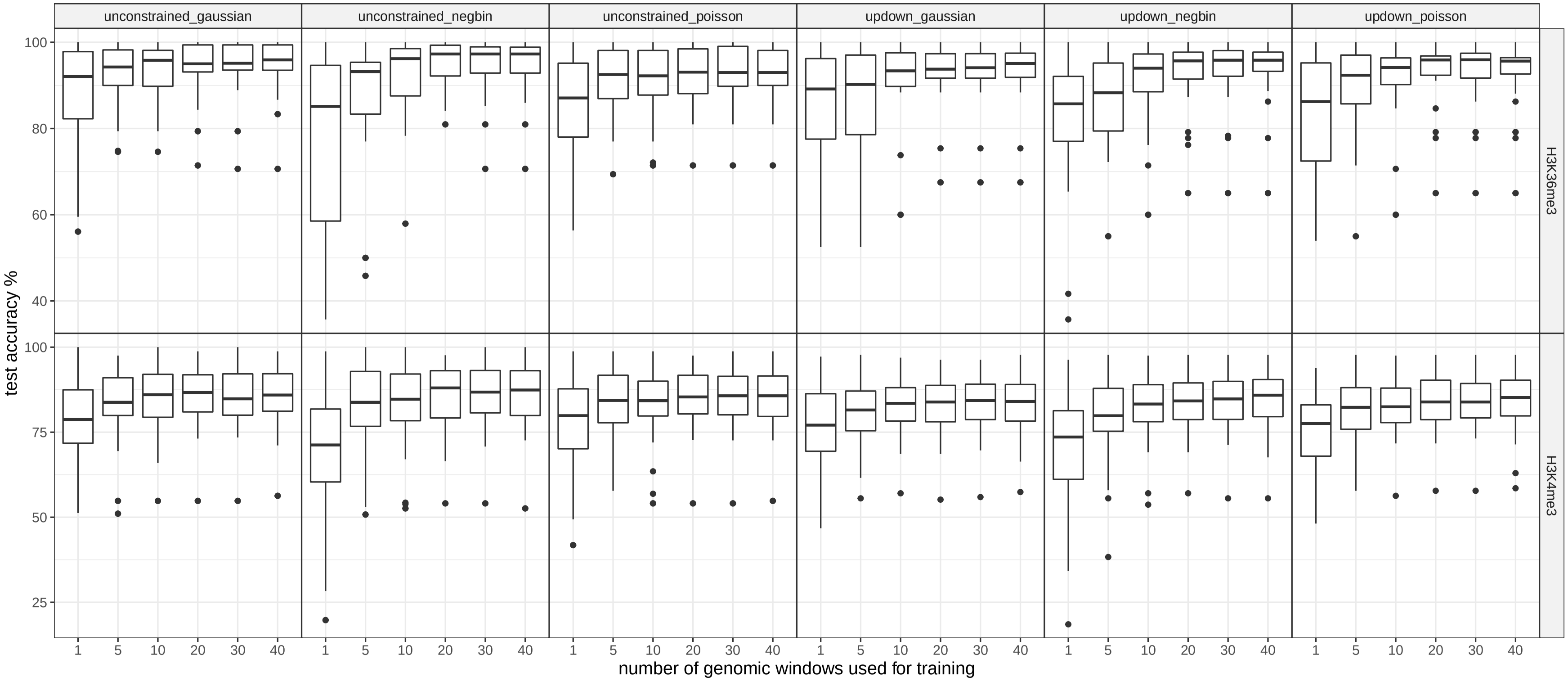

Figure 1: To determine how many labels are necessary to learn a model close to the maximum accuracy we used the cross-validation procedure described in the manuscript. Each test fold is assessed for a variable number of labeled genomic windows from the training folds (from 1 to 40). In all datasets, 10 genomic windows, with on average 5 labels per window, are enough to learn penalties and associated segmentation close to the model-specific maximum accuracy. Note that in addition to improving the average accuracy on test folds, a few genomic windows are enough to effectively reduce the variance of the results.

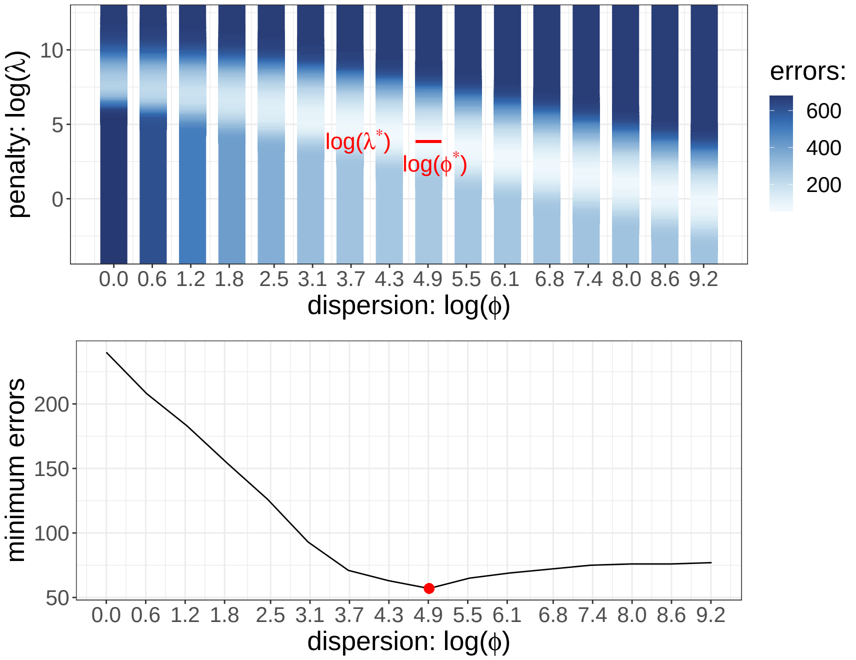

Figure 2: (**Top**) Visualization of $\sum_{m \in \text{ training set}} E_m(\phi \in \Phi, \lambda)$. The global minimum error (57), shown in red ■, is reached for $\lambda^* = 46.86$ and $\phi^* = 135.94$. (**Bottom**) For each ϕ_i, i.e 16 values evenly placed on the log scale between 1 and 10000, the minimum error of $E_m(\phi_i, \lambda)$ has been plotted. We can see the errors growing constantly at the left en right side of ϕ^* which suggests that this range of ϕ is appropriate for learning a suitable dispersion parameter value.

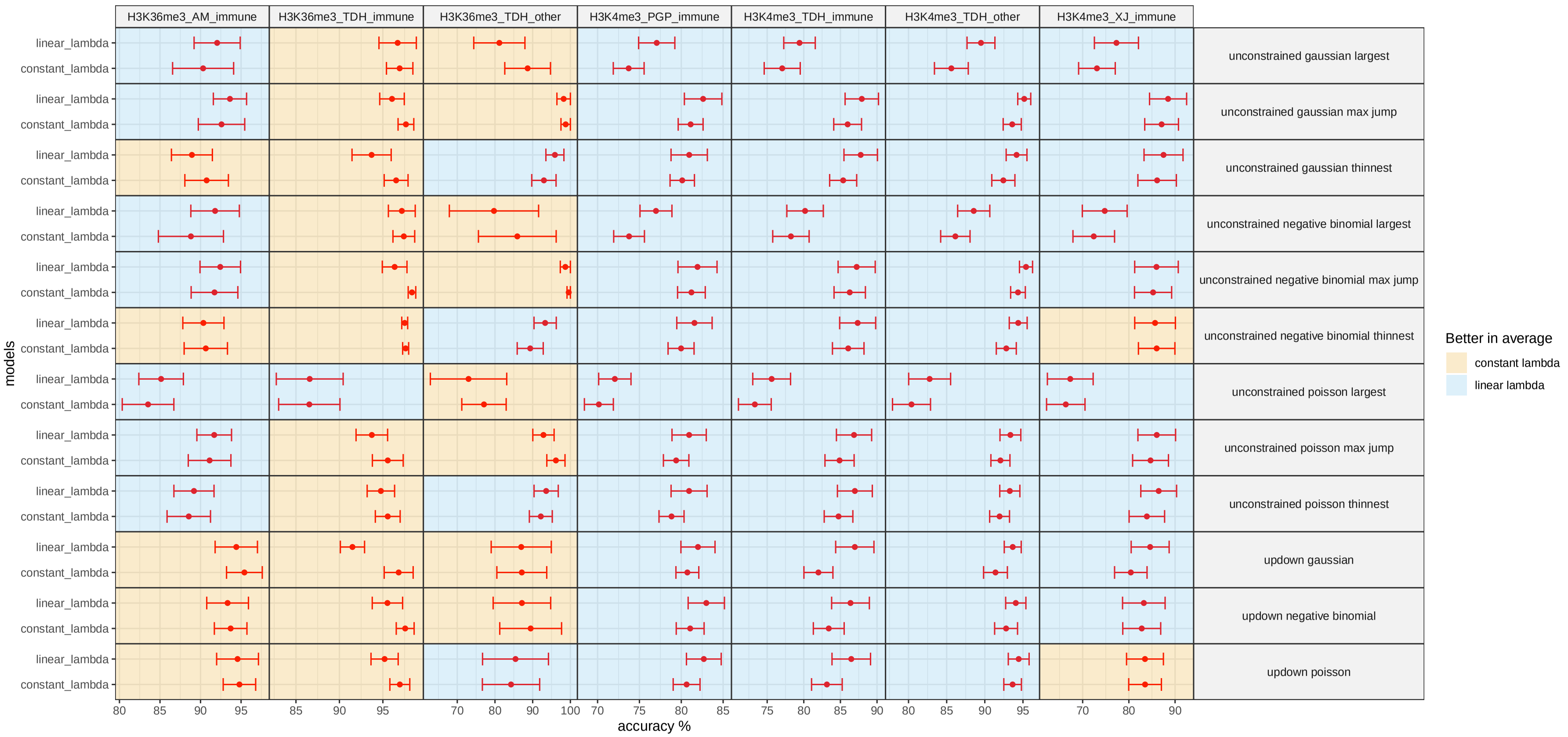

Figure 3: The *linear* λ learning method improves the accuracy of the segmentation models upon the *constant* λ learning method on H3K4me3 datasets. The mean accuracy and its 95% CI computed on the test folds is shown in red ■. In 46 of the 48 comparisons on the H3K4me3 datasets, the *linear* λ learning method was better in average than the *constant* λ learning method. After pooling the folds by type of experiment, we performed a paired t-test on each comparison. After correcting the p-values with the Benjamini & Hochberg method, 6 (/12) differences in mean accuracy were still significant (adjusted p-value < 0.05). The concerned models are: unconstrained gaussian max jump; unconstrained gaussian largest; unconstrained negative binomial largest; unconstrained poisson thinnest; updown gaussian; updown negative binomial. In H3K36me3 datasets, there is no clear trend on which learning method is the best. 24 of the 36 comparisons are in favour of the *constant* λ learning method. After pooling the folds by type of experiment and performed a paired t-test following by a correction of the p-values, none of the 12 differences in mean were significant.

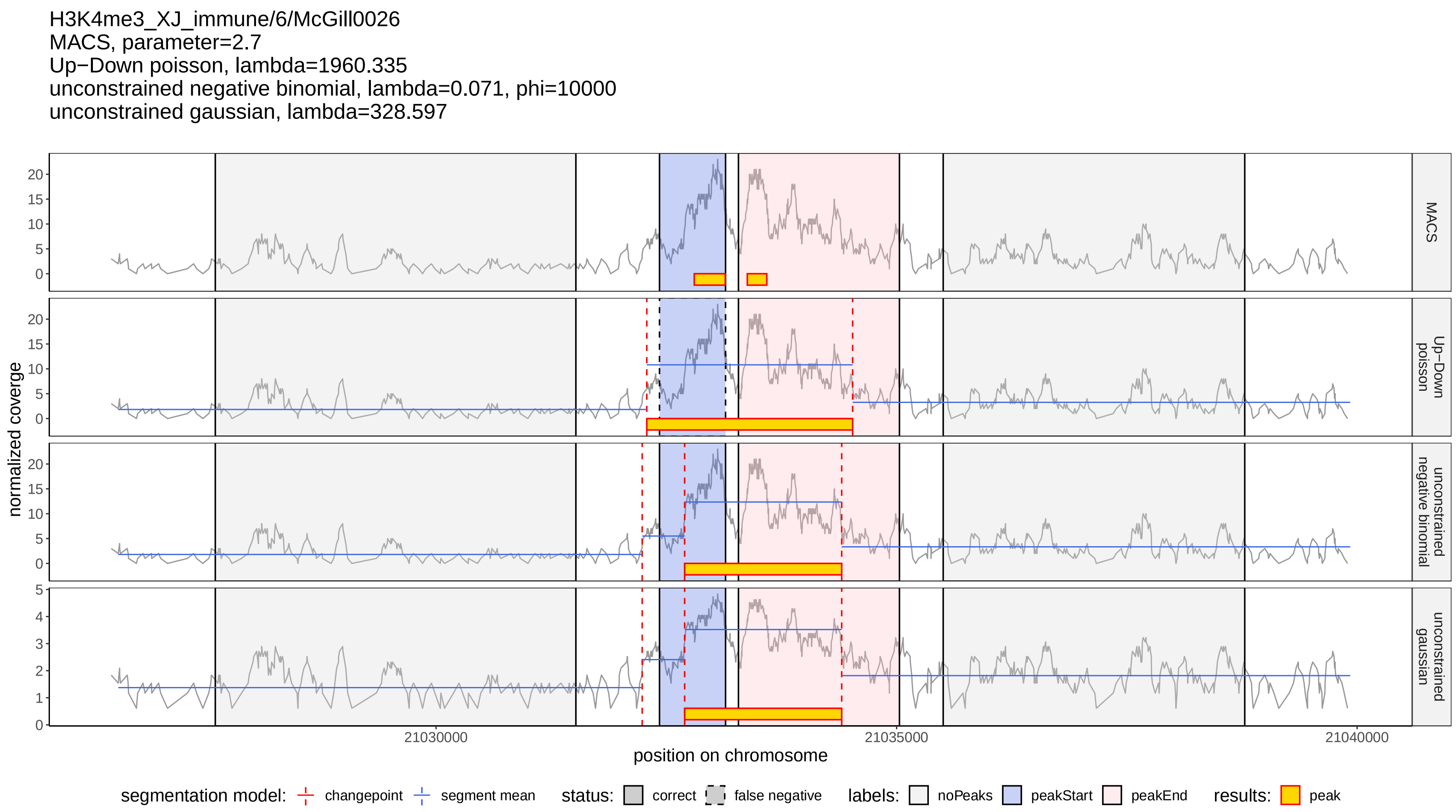

Figure 4: Visualization of the trained MACS, trained Up-Down Poisson, trained unconstrained negative binomial and trained unconstrained Gaussian models, on the normalized coverage profile (in the Gaussian case the data are additionally transformed as explained in the manuscript) from one of the biological samples (McGill0026) of the ChIP-Seq experiment directed against H3K4me3 histone marks. (**Top**) Summary of the tuning parameter values learned on the training set for each model. The parameter learned for MACS is the q-value threshold parameter.

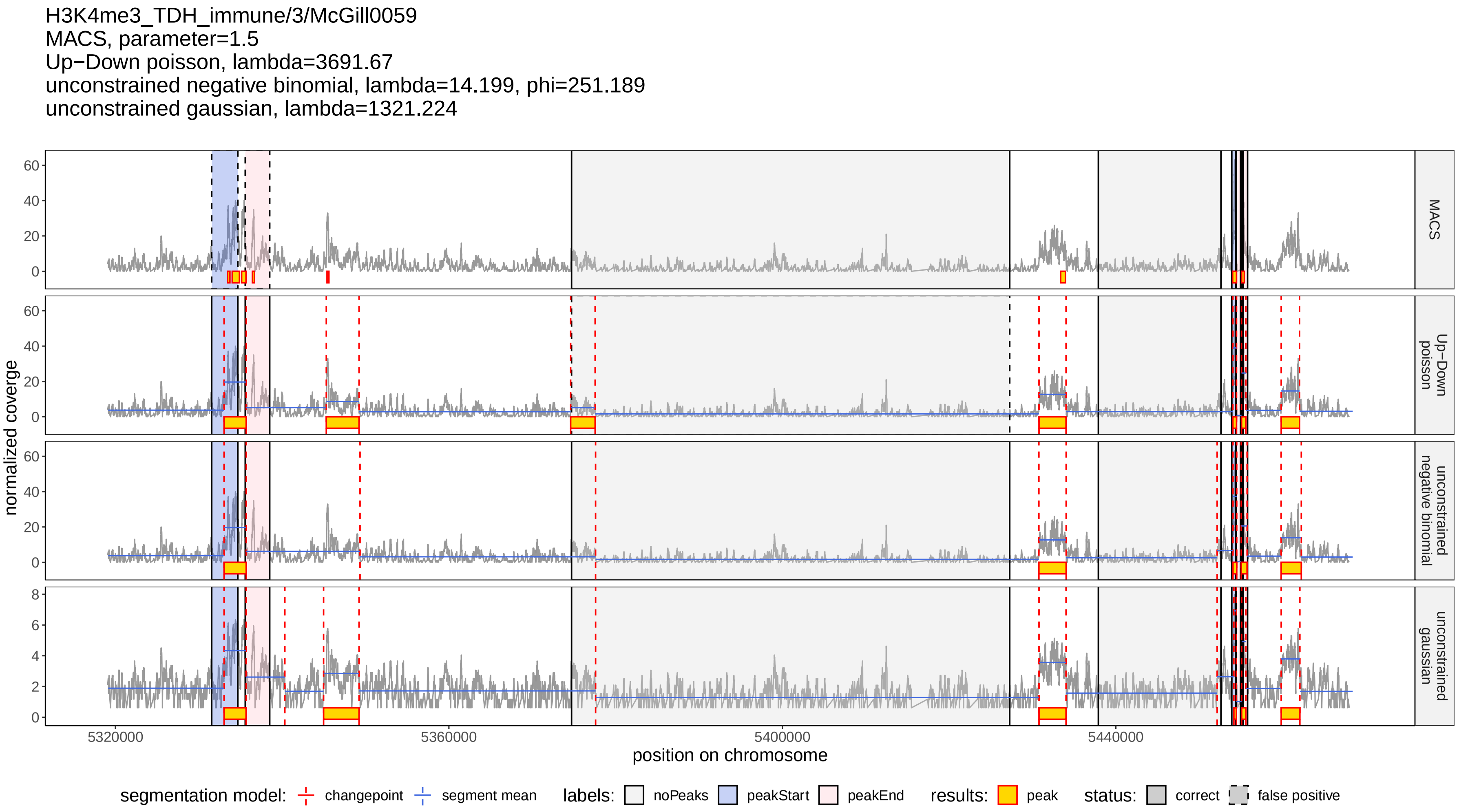

Figure 5: Visualization of the trained MACS, trained Up-Down Poisson, trained unconstrained negative binomial and trained unconstrained Gaussian models, on the normalized coverage profile (in the Gaussian case the data are additionally transformed as explained in the manuscript) from one of the biological samples (McGill0059) of the ChIP-Seq experiment directed against H3K4me3 histone marks. (**Top**) Summary of the tuning parameter values learned on the training set for each model. The parameter learned for MACS is the q-value threshold parameter.

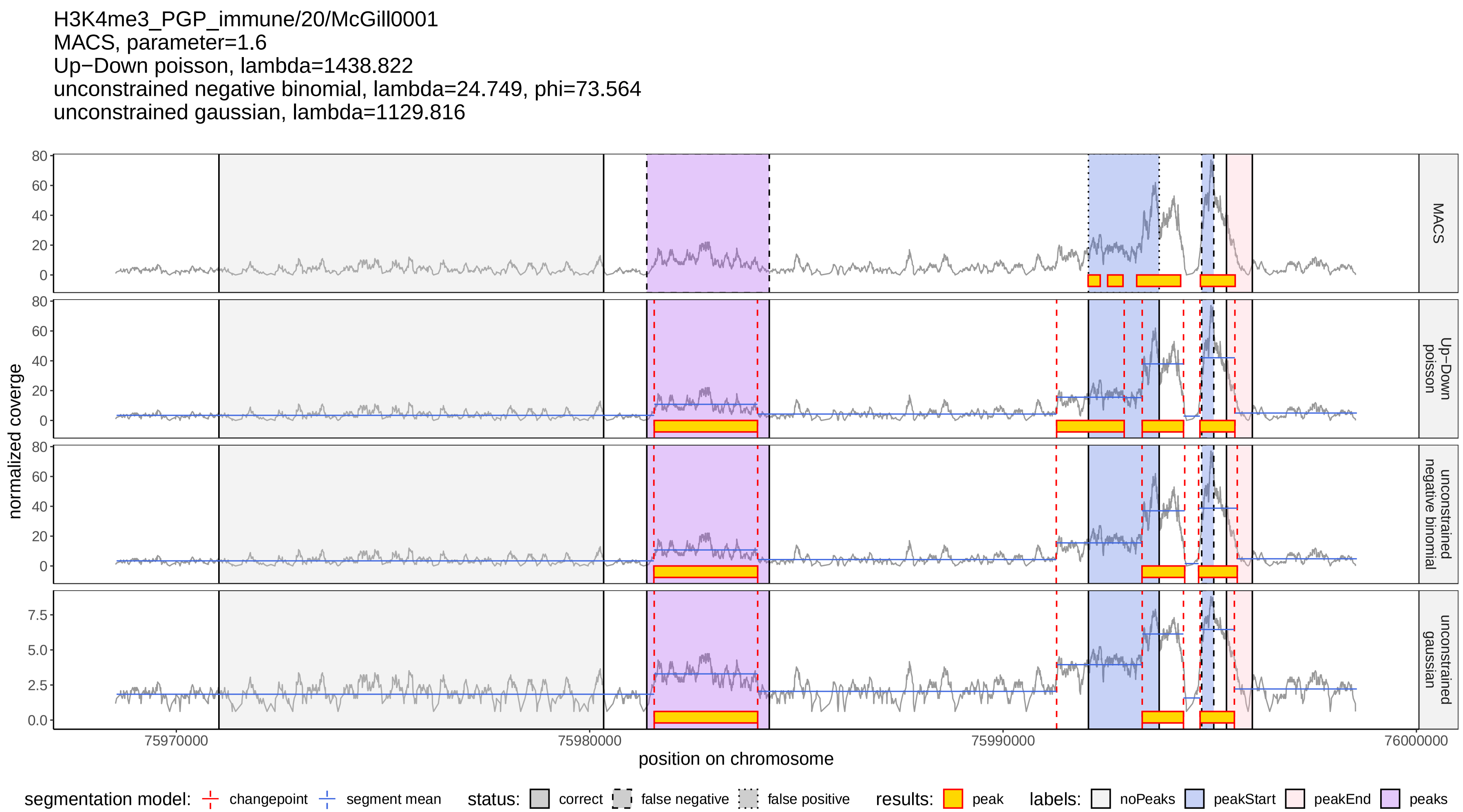

Figure 6: Visualization of the trained MACS, trained Up-Down Poisson, trained unconstrained negative binomial and trained unconstrained Gaussian models, on the normalized coverage profile (in the Gaussian case the data are additionally transformed as explained in the manuscript) from one of the biological samples (McGill0001) of the ChIP-Seq experiment directed against H3K4me3 histone marks. (**Top**) Summary of the tuning parameter values learned on the training set for each model. The parameter learned for MACS is the q-value threshold parameter.

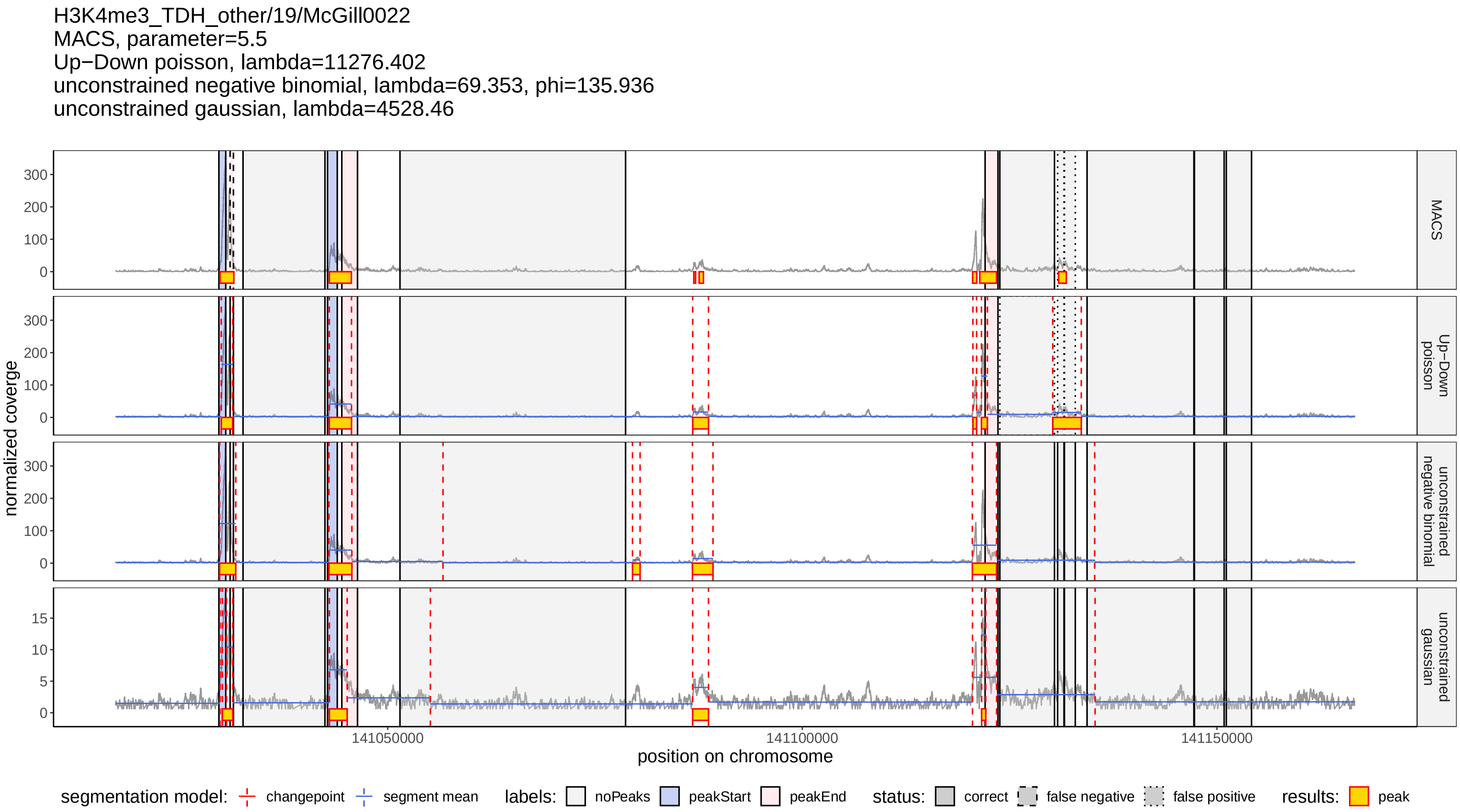

Figure 7: Visualization of the trained MACS, trained Up-Down Poisson, trained unconstrained negative binomial and trained unconstrained Gaussian models, on the normalized coverage profile (in the Gaussian case the data are additionally transformed as explained in the manuscript) from one of the biological samples (McGill0022) of the ChIP-Seq experiment directed against H3K4me3 histone marks. (**Top**) Summary of the tuning parameter values learned on the training set for each model. The parameter learned for MACS is the q-value threshold parameter.

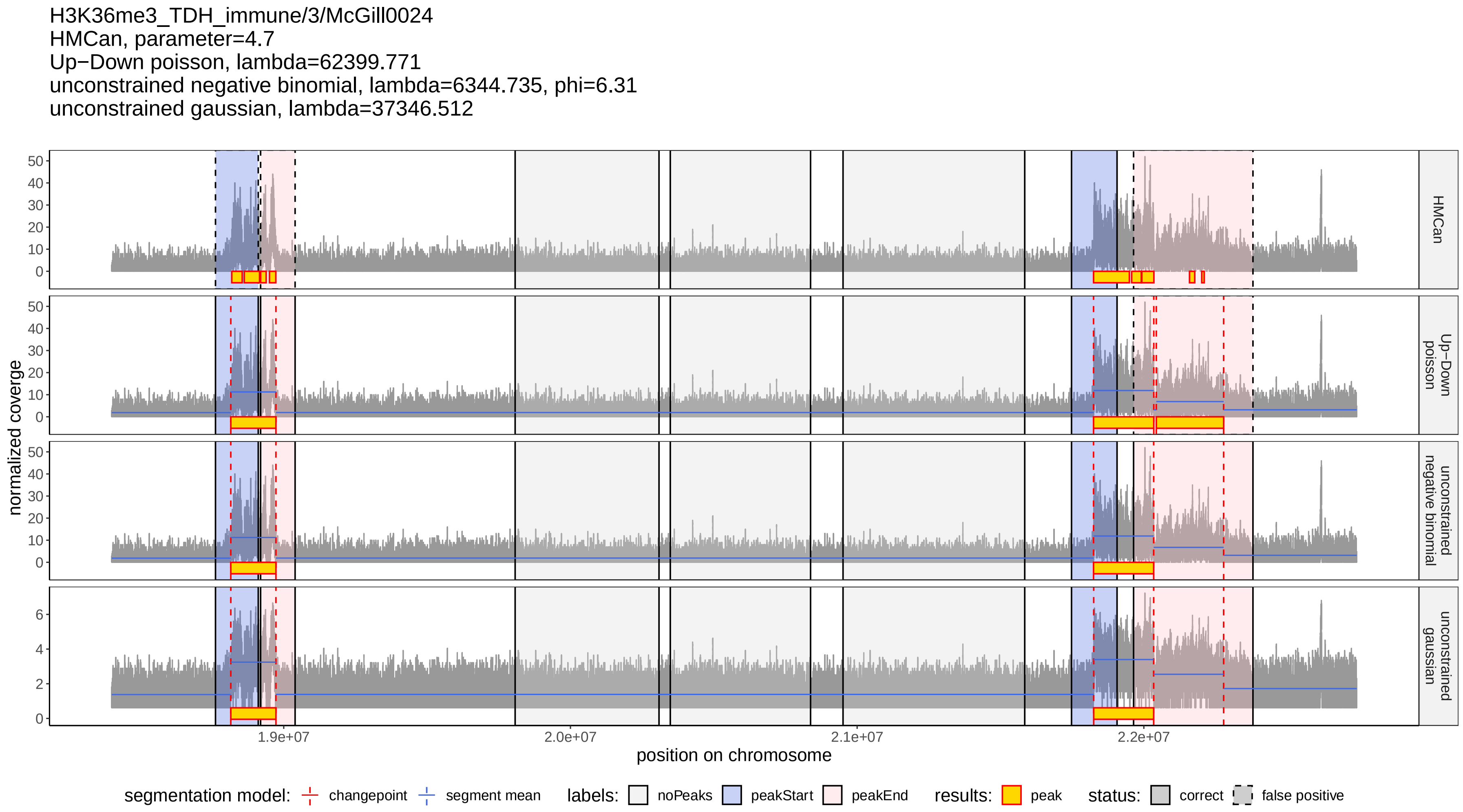

Figure 8: Visualization of the trained HMCan, trained Up-Down Poisson, trained unconstrained negative binomial and trained unconstrained Gaussian models, on the normalized coverage profile (in the Gaussian case the data are additionally transformed as explained in the manuscript) from one of the biological samples (McGill0024) of the ChIP-Seq experiment directed against H3K36me3 histone marks. (**Top**) Summary of the tuning parameter values learned on the training set for each model. The parameter learned for HMCan is the finalThreshold parameter.

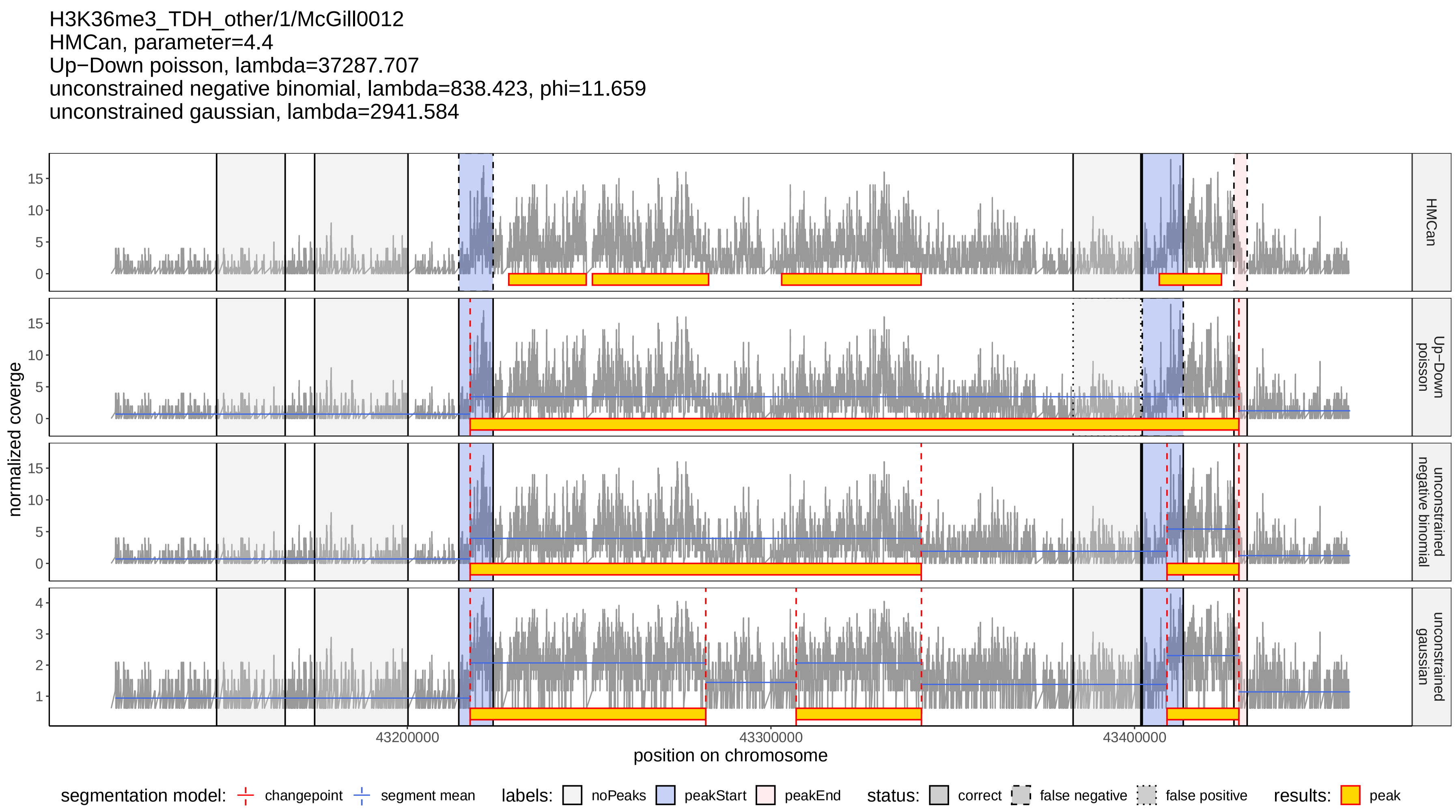

Figure 9: Visualization of the trained HMCan, trained Up-Down Poisson, trained unconstrained negative binomial and trained unconstrained Gaussian models, on the normalized coverage profile (in the Gaussian case the data are additionally transformed as explained in the manuscript) from one of the biological samples (McGill0012) of the ChIP-Seq experiment directed against H3K36me3 histone marks. (**Top**) Summary of the tuning parameter values learned on the training set for each model. The parameter learned for HMCan is the finalThreshold parameter.

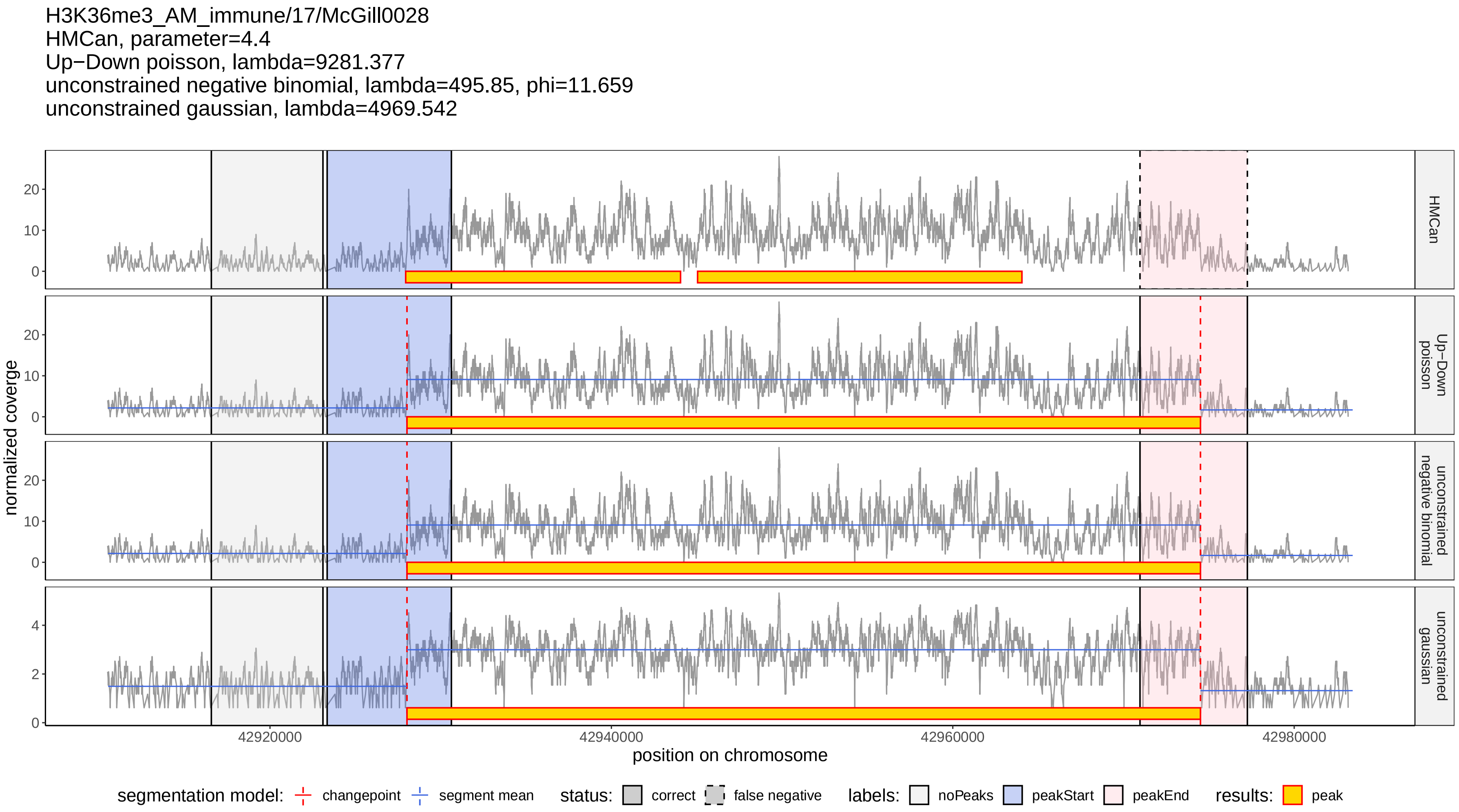

Figure 10: Visualization of the trained HMCan, trained Up-Down Poisson, trained unconstrained negative binomial and trained unconstrained Gaussian models, on the normalized coverage profile (in the Gaussian case the data are additionally transformed as explained in the manuscript) from one of the biological samples (McGill0028) of the ChIP-Seq experiment directed against H3K36me3 histone marks. (**Top**) Summary of the tuning parameter values learned on the training set for each model. The parameter learned for HMCan is the finalThreshold parameter.
